# Implementation of a Reuse Process for Liquid Crystal Displays Using an Eccentric-Form Tool

**DOI:** 10.3390/ijms10094178

**Published:** 2009-09-24

**Authors:** Pai-Shan Pa

**Affiliations:** Department of Digital Content Design, Graduate School of Toy and Game Design, National Taipei, University of Education, NO. 134, Sec. 2, Heping E. Rd., Taipei City 106, Taiwan; E-Mail:myhow@seed.net.tw

**Keywords:** liquid crystal display, nanotechnology, thin-film, eccentric-form, reuse-process

## Abstract

This study presents a new nanotechnology application involving an ITO thin-film removal reuse process using an eccentric-form negative electrode, offering a fast removal rate from the surface of liquid crystal displays (LCDs). For the precision removal process, a small amount of eccentricity of the negative electrode or a higher rotational speed of the negative electrode corresponds to a higher etching rate for the ITO. A higher flow velocity of the electrolyte and a higher working temperature also correspond to a higher removal rate. The average effect of the eccentricity is better than the effects of a pulsed current, while the current rating need not be prolonged by the off-time.

## 1. Introduction

Lighter in weight and smaller in volume, flat panel displays (FPDs) have several advantages over conventional CRT displays. Liquid crystal displays (LCDs) are the most common displays in many environments. The opportunities for use of LCD panels in notebook computers have also increased as the market has continued to grow. Panels for mobile phone displays also have excellent growth prospects [1]. After the transparent electrode layer of indium-tin oxide (ITO) is applied, the procedure is complete. With the push to increase the production capacity of LCD panels, the size of the glass substrate must increase in response to this demand [2]. Color filters are the critical components in LCDs, since each thin-film transistor (TFT) array is matched to a color filter of the same size. This means that the quality of the color filter has a decisive effect on the color reproduction of the LCD panel. The future of display technology will be in flat panel monitors, and thus TFT-LCDs will play an important role. The purpose of a display monitor is to recreate the real world in front of our eyes so that we can enjoy a visual experience with the best possible quality and the most accurate representation of information. Displays must have color in order to achieve this ideal, and it is color filters that give TFT-LCD flat panel monitors their ability to display colors [3].

Electrochemical machining (ECM) can be applied to electrolytic components [*e.g.*, silicon chips, very large scale integration (VLSI) chips and ultra large scale integration (ULSI) chips]. In ECM, a good workpiece surface quality is obtained through the optimization of the experimental conditions. The main difficulty lies in the design of the tool electrodes given the complicated process of metal removal [4]. The electrochemical technique is based on the electrochemical reaction between an electrode and a workpiece. In addition, workpiece machining through the electrochemical process can improve the precision with the appropriate control of machining conditions or the electrode geometry. Data showed that the gap width between the electrode and workpiece directly influences the current conditions and the discharge dregs of the electrolyte [5]. The experimental results of Mileham *et al*. proved that the quality of the machined surface is influenced such factors as the current density and flow rate of the electrolyte as well as the gap width in the electrochemical machining [6]. Shen used NaNO_3_ as the electrolyte to electropolish the die surface. The results showed that the surface roughness of workpieces decreases with increased current density, flow rate and concentration of the electrolyte [7]. Schuster *et al*. showed that the machining resolution can be shortened to a few micrometers by applying ultra short pulses of nanosecond duration, and microstructures can be machined by ECM [8]. In addition, workpiece machining through the electrochemical process can improve precision with the appropriate control of the machining conditions or the electrode geometry. A plate-form electrode was developed as a design tool for use in recycling systems, and good removal effects were obtained through the careful design of the experimental conditions [9].

Defects in the ITO layer are easily created during the semiconductor production processes. The primary cause of the decrease in the yield rate in LCD production is “dust”. When dust particles become attached to the LCD substrate, they impair its function, causing breaks in the circuit, short-circuits and poor performance [10–11]. This research presents a new design using electrochemical removal and an eccentric-form tool as a precision reuse process for ITO thin-film removal from the surface of liquid crystal displays.

## 2. Experimental Setup

**Figure 1.**
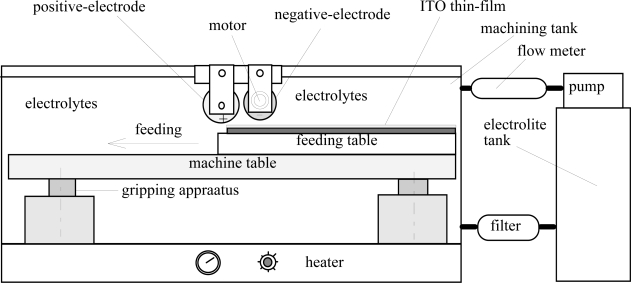
Experimental setup.

**Figure 2.**
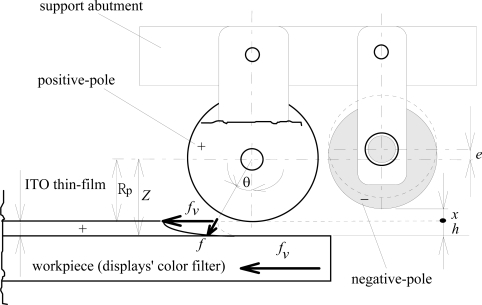
Configuration of electrodes and workpiece.

The workpiece material is a 5^th^ generation LCD panel (1,300 × 1,100 mm; 0.7 mm). The experimental set-up of the precision reuse process for ITO thin-film removal from the color filters of the display is schematically illustrated in Figure 1. The tool electrodes (including a positive electrode and a negative electrode) are shown in Figure 2. The electrolyte was NaNO_3_ with 15 wt% and PO4-3-P with 5 wt%. The amount of the reduction by removal from the color filter surface after electrochemical machining for ITO was 150 nm. The rotational speed of the negative electrode was 100 rpm. The amount of eccentricity of the negative electrode was 1, 2 and 3 mm. The gap-width between the negative electrode and workpiece was 3, 5 and 7 mm. The current rating was 50, 100, 150 and 200 A. The feed rate of the workpiece (color filter of the display) ranged from 150 to 500 mm/min. The flow rate of the electrolyte was 5, 10, 15, 20 and 25 L/min. The temperature of the electrolyte was 40, 50, 60 and 70 °C. The rotational speed of the eccentric-form negative electrode was 100, 300, 500 and 700 rpm. The pulsed period (on/off time) used 100 ms/100 ms compared with continuous direct current. The ITO layer produced was measured at more than two locations by a NanoSpec Film Thickness Measurement System (NanoSpec Film Analyzer 3000).

## 3. Results and Discussion

**Figure 3.**
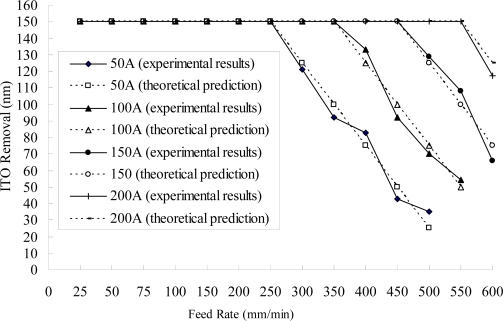
Amounts removed at different feed rates of the workpiece using different current ratings (NaNO_3_ of 10 wt% and PO4-3-P 5 wt%, 55 °C, 25 L/min, 300 rpm).

Figure 3 illustrates that an adequate removal is achieved through a combination of the current rating and the feed rate of the workpiece (the color filter of the display) for the process of electrochemical removal. At a constant current rating, the workpiece has an optimal feed for the best removal rate. A fast feed reduces the power delivered to a unit area of the workpiece surface, and a slow feed increases it. The former does not supply sufficient electrochemical power, while the latter increases the removal time and the cost. In order to achieve the same amount of removal of the ITO film (the average thickness of the ITO film was 150 nm in this study), the following combination of parameter values is suggested: 50 A and 250 mm/min, 100 A and 350 mm/min, 150 A and 450 mm/min, and 2000 A and 550 mm/min.

According to the formula of the theoretical removal rate of an alloy from Faraday’s Law [13]:

(1) $$W=\frac{\eta It}{F(\frac{n_{A}}{{\text{M}}_{A}}a_{A}+\frac{n_{B}}{M_{B}}a_{B}+....)}$$

where η is the efficiency of current, *I* is the current, *t* is time, *F* is the Faraday constant, *n_i_* is the atom number, *a_i_* is the proportion of the composition, and *M_i_* is the atomic mass.

Let *w* = *W/At* and *f* = *w/ρ*. Then:

(2) $$f=\frac{\eta I}{FA\rho (\frac{n_{A}}{M_{A}}a_{A}+\frac{n_{B}}{M_{B}}a_{B}+....)}$$

where *A* is the microelectroremoval area, ρ is the workpiece density, and *f* is the removal rate in the longitudinal direction. From the above, the theoretical feed rate of the workpiece for the same material removal rate can be calculated. Here, η, *F*, and *A* are regarded as constant for the material:

(3) $$Z=R_{p}+h$$

where *x* is the gap between the electrode and the color filters, and *h* is the removal depth of the microelectroremoval (see Figure 2):

(4) $$\text{cos}\;\theta =\frac{Z-h}{Z}=\frac{R_{P}+x}{R_{P}+x+h}$$

where *Z* is the length from the center of the positive pole to the surface of the color filters, and *R_P_* is the radius of the positive pole (see Figure 2):

(5) $$(f_{v})\;\text{sin}\;\theta =f$$

Squaring and simplifying Equations. (4) and (5), one obtains:

(6) $$h=\frac{(R_{p}+x)\;f^{2}}{2(f_{v}^{2}-f^{2})}$$

where *f_v_* is the feed velocity of the color filters, and *f* is the removal rate in the longitudinal direction. From formula (6), one obtains:

(7) $$h=\frac{\left(R_{p}+x)[\frac{\eta I}{FA\rho (\frac{n_{a}}{M_{A}}a_{A}+\frac{n_{B}}{M_{B}}a_{b}+..)}\right]}{2\{f_{v}^{2}-{\left[\frac{\eta I}{FA\rho }(\frac{n_{A}}{M_{A}}a_{a}+\frac{n_{B}}{M_{B}}a_{b}+..)\right]}^{2}\}}$$

(8) $$=\frac{(R_{P}+x){\left[\frac{\eta E\;\sigma }{Fx\rho (\frac{n_{A}}{M_{A}}a_{A}+\frac{n_{B}}{M_{B}}a_{B}+..)}\right]}^{2}}{2\{f_{v}^{2}-{\left[\frac{\eta E\;\sigma }{Fx\rho (\frac{n_{A}}{M_{A}}a_{A}+\frac{n_{B}}{M_{B}}a_{B}+..)}\right]}^{2}\}}$$

where *E* is the voltage of the gap width, and σ is the reciprocal resistance of the electrolyte.

Using Equation (7), the experimental results also agree well with the theoretical predictions (see Figure 3). Compared with the experimental results, the removal depth *h* is directly proportional to the current rating *I* and is inversely proportional to the feed rate of the workpiece (*f_v_),* which agrees well with the theoretical prediction (see Figure 3).

**Figure 4.**
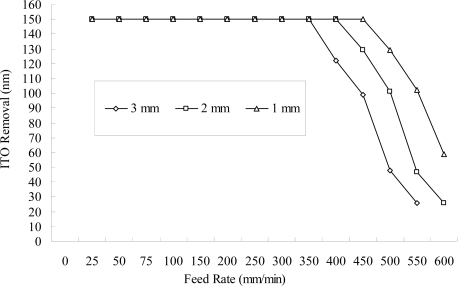
Amount removed at different feed rates and different eccentricities of the negative-electrode (NaNO_3_ of 10 wt% and PO4-3-P 5 wt%, 55 °C, 25 L/min, 100 A, continuous DC, negative-electrode 300 rpm).

**Figure 5.**
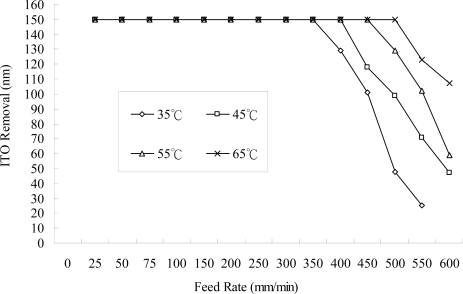
Amount removed at different feed rates of the workpiece using different temperatures of the electrolytes (NaNO_3_ of 10 wt% and PO4-3-P 5 wt%, 25 L/min, 150 A, continuous DC, negative-electrode 300 rpm).

Figure 4 illustrates that the smaller the amount of eccentricity of the negative electrode, accompanied by a small gap-width between the negative electrode and the workpiece, the less time is required for the same amount of ITO nanostructure removal since the effect of the electrochemical removal is easily developed with a sufficient supply of electrochemical power. From the experimental results, a small gap-width accompanied by a large current rating and a fast feed rate of the workpiece reduced the removal time. From Equation (8), as far as the stable operation of the electrochemical removal and dregs discharge is concerned [providing the reciprocal resistance of the electrolyte (σ) stability], the adequate amount of eccentricity of the negative electrode is 1 mm (gap width 3 mm), which is most effective in the current experiment. Figure 5 illustrates the workpiece under different temperatures of the electrolyte; the results show that a higher temperature corresponds to a higher removal rate for the ITO thin-film. One can combine a higher temperature with a fast feed rate to reduce the machining time.

**Figure 6.**
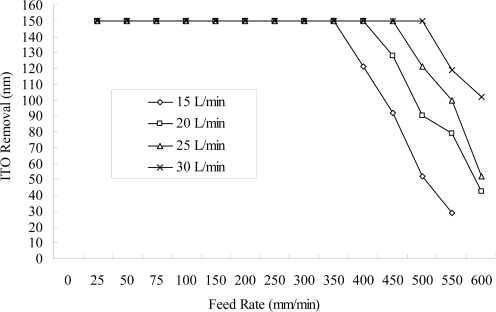
Amount removed at different feed rates of the workpiece using different flow rates of the electrolytes (NaNO_3_ of 10 wt% and PO4-3-P 5 wt%, 55 °C, 150 A, continuous DC, negative-electrode 300 rpm).

Figure 6 shows that the larger the flow rate is, the more rapid the electrolytic depositions, and as long as the heat can be conducted away, the removal rate of the ITO thin-film is improved. As a result, the use of a large electrolytic flow rate is advantageous when used with a fast feed rate of the workpiece.

**Figure 7.**
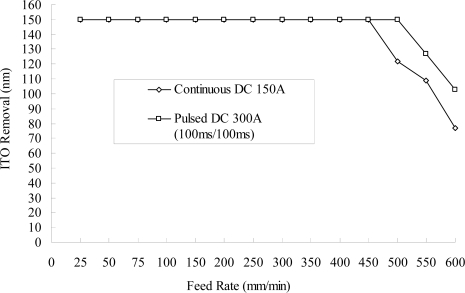
Amount removed at different feed rates of the workpiece using continuous and pulsed direct current (NaNO_3_ of 10 wt% and PO4-3-P 5 wt%, 55 °C, 150 A, negative-electrode 300 rpm).

**Figure 8.**
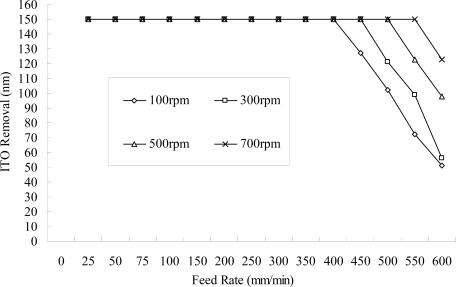
Amount removed at different rotational speeds of the negative-electrode (NaNO_3_ of 10 wt% and PO4-3-P 5 wt%, 55 °C, 150 A, continuous DC).

Figure 7 shows the effects of the pulsed direct current. In order to reach a removal amount of 150 nm for the ITO nanostructure, the same as that for the continuous direct current, the current rating needs to be proportionally increased to compensate for the off-time. It is thought that the dregs discharge during the off-time is more complete, and that it can be beneficial when associated with a fast feed rate. Figure 8 illustrates that a high rotational speed of the eccentric-form negative electrode produces high rotational flow energy and elevates the discharge mobility, which improves the removal effect. It is believed that a high rotational speed of the negative electrode is advantageous when combined with a fast feed rate of the workpiece. From Figures 7 and 8, comparing the experimental results and Equation (8) above shows that stably controlling the reciprocal resistance (σ) can stabilize the etching effect and, as a result, increase discharge mobility (providing the off-time period of the pulsed direct current or a large electrode rotational speed), guide discharge transport and provide flushing passage to provide reciprocal resistance (σ) stability.

## 4. Conclusions

Through the ultra-precise removal of ITO nanostructures, the semiconductor industry can effectively reuse defective products, which reduces production costs. For the removal process, a higher current rating with a faster feed rate of the liquid crystal display effectively achieves fast removal. A pulsed direct current can improve the dregs discharge and complement a fast feed rate, but it increases the current rating. A small amount of eccentricity in the negative-electrode accompanied by a small gap-width requires less time for the same amount of ITO removal. A higher rotational speed of the negative-electrode, a higher flow velocity of the electrolyte, or a higher working temperature corresponds to a higher removal rate for the ITO nanostructure.
